# WHAT FACTORS CONTRIBUTE TO DELAYED GASTRIC EMPTYING AFTER DUODENOPANCREATECTOMY WITH PILORIC PRESERVATION?

**DOI:** 10.1590/0102-672020210002e1592

**Published:** 2021-10-18

**Authors:** Ricardo Tadashi NISHIO, Adhemar Monteiro PACHECO-JR, André de MORICZ, Rodrigo Altenfelder SILVA

**Affiliations:** 1 Department of Surgery, Faculty of Medical Sciences, Santa Casa de São Paulo, São Paulo, SP, Brazil

**Keywords:** Gastric emptying, Pancreas, Pylorus, Pancreaticoduodenectomy, Postoperative complications, Esvaziamento gástrico, Pâncreas, Piloro, Pancreaticoduodenectomia, Complicações pós-operatórias

## Abstract

**Background:**

The delay in gastric emptying is the second most frequent complication after duodenopancreatectomy with pyloric preservation, that increases hospitalization time and hospital costs.

**Aim:**

To identify factors that contribute to the appearance the delay in this surgical procedure.

**Method:**

Ninety-five patients were submitted to duodenopancreatectomy with pyloric preservation. After retrospective analysis of the medical records, it was observed that 60 had prolonged hospitalization due to complications. Thus, univariate and multivariate logistic regression were used to analyze predictors of delayed gastric emptying.

**Results:**

Delay was present in 65% (n=39) and pancreatic fistula in 38.3% (n=23). Univariate analysis revealed that the presence of pancreatic complications (pancreatic fistula, p=0.01), other intracavitary complications with the appearance of abdominal collections (p=0.03) and hypoalbuminemia (p=0.06) were responsible, also confirmed by the multivariate analysis. In those who presented delay without a determined cause, it was observed that high levels of total bilirubin (p=0.01) and direct bilirubin (p=0.01) could be related to it.

**Conclusion:**

The delay in gastric emptying in patients undergoing duodenopancreatectomy with pyloric preservation is due to intracavitary complications.

## RESUMO

**Racional:**

O retardo do esvaziamento gástrico é a segunda complicação mais frequente após a realização da duodenopancreatectomia com preservação pilórica, aumentando o tempo de internação e custos hospitalares.

**Objetivo:**

Identificar fatores que contribuem para o aparecimento desse retardo nesse procedimento cirúrgico.

**Método:**

Noventa e cinco doentes foram submetidos à duodenopancreatectomia com preservação pilórica. Após análise retrospectiva dos prontuários observou-se que 60 apresentaram internação prolongada por complicações. Assim, utilizou-se a regressão logística uni e multivariada para análise de fatores preditores do retardo.

**Resultados:**

O retardo esteve presente em 65% (n=39) e a fístula pancreática em 38,3% (n=23). A análise univariada revelou que a presença de complicações pancreáticas (fístula pancreática, p=0,01), outras complicações intracavitárias com aparecimento de coleções abdominais (p=0,03) e hipoalbuminemia (p=0,06) foram os responsáveis, resultados estes também confirmados pela análise mutilvariada. Naqueles que apresentaram retardo sem causa determinada, observou-se que níveis elevados de bilirrubina total (p=0,01) e bilirrubina direta (p=0,01) poderiam estar relacionados a ele.

**Conclusão:**

O retardo do esvaziamento gástrico nos pacientes submetidos à duodenopancreatectomia com preservação pilórica é decorrente de complicações intracavitárias.

## INTRODUCTION

In high-volume centers for pancreatic operations, mortality rates are less than 5%^29^. However, complications are frequent and can happen in up to 73%^19^ ^,^ ^21^. Delayed gastric emptying (GED) is defined as the patient’s inability to tolerate the oral diet until the end of the first week after pancreatic surgery^29^. It is a relatively common complication after pancreaticoduodenectomy and may occur in up to 40%^12^. Despite the low mortality rate resulting from it, its occurrence is associated with prolonged hospital stay and high costs^1^.

GED occurs after pyloric-sparing duodenopancreatectomy (PPPD), in the Whipple procedure and in distal pancretectomies^16^. PPPD was described by Traverso and Longmire^25^ in 1978, being initially used in the treatment of chronic pancreatitis and, later, in the treatment of periampullary neoplasms.

The aim of this study was to identify factors related to GED after PPPD.

## METHODS

This work was carried out by the Biliary Ducts and Pancreas Group, Department of Surgery, Faculty of Medical Sciences, Santa Casa de São Paulo, São Paulo, SP, Brazil, approved by the Ethics and Research Committee of the Irmandade da Santa Casa de São Paulo, São Paulo, SP, Brazil, registered in Plataforma Brasil (CAEE 7127087.2.0000.5479) and was conducted according to Declaration of Helsinki.

**FIGURE 1:**
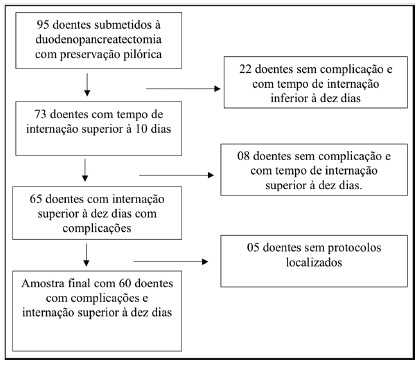
Selection of patients undergoing PPPD

From January 2001 to December 2016, all patients undergoing PPPD were included. Those whose protocols and medical records were filled out incorrectly were excluded. During the period analyzed, 95 patients underwent PPPD. According to the postoperative protocol, those who evolved without postoperative complications were discharged from the hospital until the 9^th^ day. Of the 95 initial patients, 73 had been hospitalized for 10 days or more. Of these, eight had no postoperative complications and five had incorrectly filled out protocols and medical records. Thus, sample included 60 patients with postoperative complications (Figure 1).

In the present study, complications rates, mortality and reoperation of PPPDs performed in the period were studied. In addition, it was decided to also study which complications were present in the postoperative period. To assess GED, the association of preoperative, intraoperative and postoperative data was chosen, such as gender, age, disease type operated by the PPPD technique, surgical time, amount of packed red blood cells transfused during the operation, preoperative exams (hemoglobin, total bilirubin and fractions, alkaline phosphatase, gamma-glutamyltransferase, urea, creatinine, albumin) and occurrence of pancreatic postoperative complications, such as fistula, acute pancreatitis and others that determined the appearance of intraoperative abdominal collection, as biliary fistula, enteric fistula, hemorrhage and abscesses.

The GED after pancreatectomy can also be divided into primary and secondary. The primary is manifested in the absence of other intracavitary complications. The secondary is the result of other intra-abdominal complications such as fistulas and collections that induce gastroparesis and inability to tolerate the oral diet offered to the patient.

For the evaluation and comparison of the primary and secondary GED, the operative time, the amount of transfused red blood cell concentrate and the preoperative values ​​of blood tests were studied, such as the values ​​of hemoglobin, total bilirubin and fractions, alkaline phosphatase, gamma-glutamyltransferase , albumin, urea and creatinine.

### Statistical analysis

IBM® SPSS^®^ Statistics for Windows, version 21.0, IBM Corporation, Armonk, New York, USA and SigmaStat for Windows, version 3.5, Systat Software, San Jose, California, USA were used and p-value equal to or less than 5% (p≤0.05) was adopted, except for the univariate analysis, in which p equal to or less than 10% (p≤0.1) was adopted. Data that showed statistical significance in the univariate analysis were analyzed using the multivariate regression method, Backward Stepwise Likelihood Ratio model. To compare proportions, the chi-square test was used. For the description of quantitative data, it was verified if they presented normal distribution. For data with normal distribution, the mean and standard deviation were used. For data that did not present a normal distribution, the median and interquartile range (25%-75%) were used. Normal distribution was analyzed using the Shapiro Wilk test and to compare numerical variables the Mann-Whitney test was used.

## RESULTS

From January 2001 to December 2016, 95 PPPDs were held. Sixty patients had postoperative complications with a hospital stay of more than 10 days. Of them, 25 (41.6%) were women and 35 (58.4%) men, with an average length of stay of 24.9 days.

PPPDs were performed for the treatment of the following diseases: adenocarcinoma of the duodenal papilla (n=30, 50%), adenocarcinoma of the pancreas (n=20, 33.3%), distal cholangiocarcinoma (n=8, 13, 3%) and chronic pancreatitis (n=2, 3.4%).

Postoperative complications were present in 66.7% (n=60) of all 90 PPPDs performed; the mortality rate in the period was 23.3% (n=21) and the reoperation rate was 33.3% (n=30).

Among the postoperative complications, pancreatic fistula was present in 23 cases (38.3%) and GED in 39 (65%). Bleeding occurred in 11.7% (n=7), enteric fistula in 6.7% (n=4), acute pancreatitis in 5% (n=3) and two patients (3.3%) had vascular complications related to thrombosis of the superior mesenteric vein and hepatic artery. In 10 patients, extra-abdominal complications such as pulmonary embolism, pneumonia and acute myocardial infarction were reported.

GED was observed in 39 patients. In 32, it was associated with another intra-abdominal complication, with pancreatic fistula being the most common complication present (n=17). In seven it was unrelated to any other complication.

Univariate analysis revealed that GED was not related to gender (p=0.71) and age. Furthermore, it was not related to the type of disease operated, such as pancreatic adenocarcinoma (p=0.59) and other periampullary neoplasms (p=0.63). Surgical time (p=0.33), intraoperative transfusion (p=0.26), preoperative hemoglobin (p=0.39), alkaline phosphatase (p=0.52), gamma-glutamyltransferase (p=0.31), urea (p=0.35) and creatinine (p=0.86) were not associated with the appearance of GED. This analysis also revealed that there is association with pancreatic complications, such as fistula and acute pancreatitis (p=0.01), in the presence of other intra-abdominal complications that determine collection (p=0.03), such as enteric fistula, hemorrhage, vascular complications and in the presence of hypoalbunemia (p=0.06, Table 1). 

Multivariate analysis confirmed that the presence of pancreatic complications (p=0.005), other complications that determine intra-abdominal collection (p=0.02) and hypoalbuminemia (p=0.02) were related to the onset of GED in the postoperative (Table 2). 

**TABLE 1:** Postoperative GED after pyloric-sparing duodenopancreatectomy (n=39): univariate logistic regression*

Variable	n	OR	IC (95%)	p
Gender (F=15 M=24)	39	0.81	0.27-2.41	0.71
Age	39	0.98	0.93-1.03	0.49
Pancreas neoplasm	13	0.71	0.2-2.44	0.59
Neoplasm of distal bile duct and duodenal papilla	23	0.66	0.12-3.57	0.63
Operating time (min)	39	1	0.99-1.01	0.33
Red blood cell concentrate (number of bags)	39	0.75	0.44-1.27	0.26
Hemoglobin (g/dl)	39	1.16	0.82-1.63	0.39
Total bilirubin (mg/dl)	39	1.01	0.96-1.07	0.53
Direct bilirubin (mg/dl)	39	1.03	0.96-1.11	0.31
Alkaline phosphatase (U/l)	39	1	0.99-1	0.52
Gamaglutamyltransferase (U/l)	39	1	0.99-1	0.31
Urea (mg/dl)	39	1.02	0.97-1.06	0.35
Creatinine (mg/dl)	39	1.02	0.76-1.38	0.86
Albumin (g/dl)	39	2.44	0.93-6.39	0.06*
pancreatic complications	20	7.77	1.52-39.75	0.01*
Other intra-abdominal complications with collection	10	5.66	1.12-28.45	0.03*

*Backward stepwise likelihood ratio model (p=0.1)

**TABLE 2:** Postoperative GED after pancreaticoduodenectomy with pyloric preservation (n=39): multivariate logistic regression*

Variable	n	OR	IC (95%)	p
Pancreatic complications	20	20.6	2.45-171.77	0.01
Other intra-abdominal complications with collection	10	11	1.458-82.98	0.02
Albumin (g/dl)	26	23.3	1.16-10.20	0.03

*Backward stepwise likelihood ratio model (p=0.5)

Comparing patients with primary and secondary GED through the chi-square test, it was found that the presence of pancreatic complications and other complications with intracavitary collection are more favorable to the appearance of GED with a risk of 6.27 and 11, 48, respectively, higher when compared to the primary GED.

**FIGURE 2:**
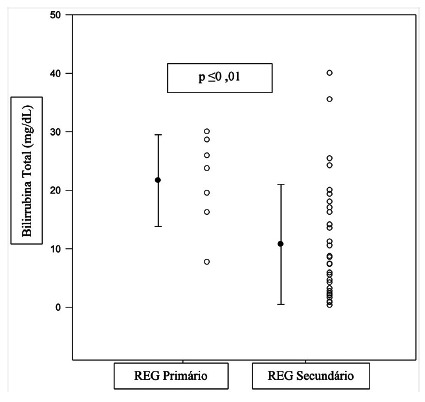
Comparison between preoperative levels of total bilirubin that presented GED after PPPD (n=39)

**FIGURE 3:**
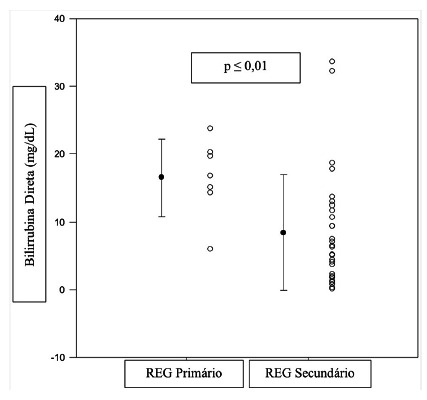
Comparison between preoperative levels of direct bilirubin with GED after PPPD (n=39)

In patients with primary GED, the duration of the surgical procedure (p=0.87), blood transfusion (p=0.80), preoperative hemoglobin level (p=0.38), alkaline phosphatase (p=0.81), gamma-glutamyltransferase (p=0.19), urea (p=0.18), creatinine (p=0.49) and albumin were not related to its appearance. However, it was found that total (p=0.01) and direct (p=0.01) bilirubin levels were indeed related when compared to patients with secondary GED (Figures 2 and 3).

## DISCUSSION

GED is a frequent complication of pancreatoduodenectomy and can be found in up to 40% of all pancreatic resections^12^. It is defined as the patient’s inability to tolerate the oral diet seven days after the surgical procedure. It is classified into grades A, B and C according to the need to use a nasogastric tube, the clinical conditions, use of prokinetic drugs and enteral or parenteral diet^29^. Stomach scintigraphy is the exam of choice for confirmation^9^. However, this test is rarely available in hospitals, and the diagnosis is made through the clinical findings. Despite the low mortality of this complication, the management of GED requires specific care such as the use of special diets, prokinetic drugs such as metoclopramide and, less frequently, surgical procedures or interventional radiology^9^. As a result, hospitalizations due to it are prolonged with increased hospital costs^1^.

In 1978, Traverso and Longmire^25^ described PPPD for the treatment of chronic pancreatitis. Over time, this technique has been used in the treatment of pancreatic adenocarcinoma, cholangiocarcinoma and other periampullary neoplasms. Both PPPD and the Whipple operation have similar mortality and morbidity rates and there is no difference in quality of life between these two procedures^24^. As demonstrated by Parmar et al^16^, GED can manifest itself after all types of pancreatic procedures, mainly PPPD.

As with all surgical procedures, pancreatoduodenectomy is susceptible to postoperative complications, which can occur in up to 73%^21^. This study revealed a high rate of complications and mortality. This can be explained by the group of patients selected and by the characteristics of the service: a teaching hospital with residents and surgeons at different stages of surgical training. Patients who experienced complications, for the most part, underwent PPPD for the treatment of adenocarcinoma of the papilla. It is known this procedure for these cases present more complications because papillary adenocarcinoma causes earlier jaundice, with a thinner hepatic duct and pancreas of normal consistency and thin duct, resulting in higher fistulization rate^17^. In addition, it is a low-volume center that serves the public health system, treating patients who are often unable to perform post-operative care. As mentioned in other publications, hospitals with surgeons at different stages of surgical learning may have more complications and higher mortality^21^ ^,^ ^26^. Another consideration to be made is the fact that the mortality rate reflects a 15-year period that coincides with the beginning of the PPPD standardization in our service, not reflecting the current mortality rate. It is known that after correct standardization and technological acquisition, mortality after pancreatoduodenectomy may be less than 2%^13^. It is important to note that the concept of high mortality associated with low-volume centers has changed. It has been proven that it is not related to the volume of patients operated, but to the delay in diagnosis and treatment of complications^27^.

As demonstrated in this study, GED is associated in most cases with other intra-abdominal complications that determine collection. This fact has already been demonstrated by Robinson et al^20^ in a previous study with 416 patients; also revealed that BMI above 35 is related to GED, a factor not studied here.

Another relevant fact in this study was the association of preoperative hypoalbunemia with GED. This reflects the poor nutritional status of patients, characteristic of the population served by the public health system in cancer cases. As a result, there is a greater tendency for the appearance of fistulas and anastomotic dehiscence, as evidenced in other publications that found a higher incidence of complications and prolonged hospitalization in patients with malnutrition^8^ ^,^ ^15^.

Initially, the PPPD was associated with the GED. However, in 2013 Parmar et al^16^, in a study with 711 cases, observed that GED occurred both in the Whipple operation and in the PPPD. In addition, they associated with postoperative pancreatic fistula, sepsis, local infection and the need for reoperation or radiological intervention. It is understandable that severe sepsis or worsening of underlying clinical disease may contribute to GED due to the need to use vasoactive drugs that promote vasoconstriction and ischemia.

Although the GED pathophysiology is not fully understood, some theories such as ischemia, edema or nerve damage after dissection have been postulated. In this study, patients who presented GED without any other intra-abdominal complications had higher levels of total and direct bilirubin when compared to those with secondary GED. Therefore, it can be assumed that bilirubin may have deleterious effects that contribute to it. Mendez Sanchez et al^11^ studied the deleterious effects of elevated bilirubin in patients with Gilbert’s syndrome and found that patients with it had GED when compared to normal patients, thus being possible to relate GED with elevated levels of bilirubin.

As it is a complication frequently associated with other intracavitary complications, the attempt at prevention seems to be a strategy to be adopted.

Pancreatic fistula is the most frequent complication after pancreaticoduodenectomy^2^. It is defined as a deficiency in the healing of the pancreatojejunal anastomosis or leakage of secretion from the pancreatic parenchyma. As it is the most common complication, avoiding it is the strategy to follow. In a review, Søreide et al^23^ associated fistula with acute pancreatitis, complication of underlying diseases, malnutrition and surgical site infection. This study revealed the development of GED as a consequence of malnutrition followed by fistulas and formation of intra-abdominal collections.

As GED is a non-negligible complication, different strategies were developed in its prevention. In 2013, Imamura et al^6^ compared the precolic and transmesocolic duodenojejunal anastomosis, finding no differences between them regarding GED. Later, this study was corroborated by others that also did not show differences between them^3,^ ^5^. In 2016, Imamura et al^6^ also compared the precolic duodenojejunal anastomosis with the transmesocolic anastomosis, verifying that the precolic anastomosis has a lower rate of GED. The rationale for this finding is the fact that the precolic anastomosis is less influenced by secretions from the pancreatic fistula, resulting in lower GED. Shimura et al^22^ compared the traditional PPPD with that using stomach uprighting, precolic duodenojejunal anastomosis, internal drainage of the pancreatojejunal anastomosis and making an omentum patch over this last anastomosis. In this technical modification, he did not observe the occurrence of pancreatic fistula and GED.

In Brazil, PPPD is used in many services for the treatment of periampullary neoplasms. The first attempt to minimize complications is the result of the work of Machado et al^10^ who proposed performing pancreatojejunal and hepaticojejunal anastomoses in two separate loops in Roux-in-Y

**FIGURE 4:**
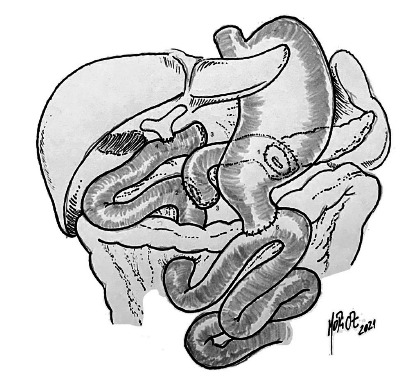
Reconstruction after pancreatoduodenectomy with pyloric preservation: 1) pancreatojejunal anastomosis; 2) enteroenteric anastomosis; 3) modified Kenneth Warren); 4) hepaticojejunal anastomosis); 5) duodenojejunal anastomosis (Source: Group of Biliary Ducts and Pancreas, Department of Surgery, Faculty of Medical Sciences, Santa Casa de São Paulo - illustration

In the Biliary Ducts and Pancreas Group of the Faculty of Medical Sciences of Santa Casa de São Paulo, PPPD is the standard procedure for the treatment of periampullary neoplasms, with the Whipple operation being reserved for selected cases. Differently from the technique proposed by Machado et al^10^, we performed the pancreatojejunal anastomosis and the hepaticojejunal anastomosis in two separate loops using the modified Kenneth Warren technique, that is, we performed the section of the loop with a linear stapler. The section of the loop in Kenneth Warren is justified by the previous findings of Pacheco&Fava^14^ on the recanalization of the ligature of the loops in Kenneth Warren. Another advantage of this modification is the lack of a mesenteric section to make the Roux-in-Y loop. Finally, we performed the precolic duodenojejunal anastomosis and drainage in the cavity. At the end of this anastomosis, a nasoenteral tube is placed for postoperative feeding (Figure 4).

Other techniques have been proposed to reduce the incidence of GED, such as pylorectomy. Studies on this procedure are divergent and there are no benefits of pylorus resection on its preservation^30^. It can be seen, therefore, that GED is not only related to PPPD and that pyloric preservation is not a risk factor for its development.

As the primary GED occurred in a small number of patients with higher bilirubin levels, it is important to discuss preoperative biliary drainage. Findings on this subject are divergent; one study reported a lower rate of GED when preoperative biliary drainage was performed^18^. However, others revealed that it was not prevented by performing biliary drainage and with an increase in the rate of infection^4^. We believe that biliary drainage should be performed in selected cases, such as in the presence of acute cholangitis, liver failure with impaired coagulation, and renal failure. Although we report that higher levels of bilirubin may be related to GED, the number of patients who presented this finding is still small.

For a better evaluation, PPPDs should have been compared with the Whipple operation. However, in our service this procedure is performed only in selected cases.

## CONCLUSION

Delayed gastric emptying is not related to the PPPD technique. It is a consequence of other intra-abdominal complications that determine collection, especially pancreatic fistula.
